# High-Dose Interleukin-2 (HD IL-2) Therapy Should Be Considered for Treatment of Patients with Melanoma Brain Metastases

**DOI:** 10.1155/2013/726925

**Published:** 2013-05-13

**Authors:** Melinda B. Chu, Mark J. Fesler, Eric S. Armbrecht, Scott W. Fosko, Eddy Hsueh, John M. Richart

**Affiliations:** ^1^Department of Dermatology, Anheuser-Busch Institute, Saint Louis University, 1402 S. Grand Boulevard., 4th Floor, St. Louis, MO 63104, USA; ^2^Division of Hematology and Oncology, Saint Louis University, 3655 Vista Avenue, 3rd Floor, St. Louis, MO 63110, USA; ^3^Center for Outcomes Research, Saint Louis University, 3545 Lafayette Avenue, 4th Floor, St. Louis, MO 63104, USA; ^4^Division of General Surgery, Saint Louis University, 3655 Vista Avenue, St. Louis, MO 63110, USA

## Abstract

A retrospective review was performed on patients with stable melanoma brain metastases treated with HD IL-2 therapy (720,000 IU/kg per dose intravenously; 14 doses, 2 cycles per course, maximum 2 courses) from January 1999 to June 2011 at Saint Louis University. There were 5 men and 3 women; median age was 52.2 years (26.8–61.1 years). One patient started treatment with lung lesions only (after resection of melanoma brain disease) and experienced partial response. Seven patients had brain metastases at treatment initiation. Median overall survival (mOS) for entire cohort (*n* = 8) was 8.7 months (2.1 to 19.0 months). All patients with brain metastases at first dose (*n* = 7) showed progressive disease; mOS was 6.7 months (range 2.1–18.2 months) for this group. Patients received radiosurgery and whole brain radiation before and after HD IL-2 therapy. One patient had symptoms suggestive of neurotoxicity. A history of alcohol abuse was revealed during admission. The patient's symptoms improved with initiation of an alcohol withdrawal protocol. In this analysis, patients with melanoma brain metastases received HD IL-2 without treatment-related mortality. We think that HD IL-2 should be considered as a treatment option in patients with melanoma brain metastases who are otherwise eligible for therapy.

## 1. Introduction

High-dose interleukin-2 (HD IL-2) therapy was FDA approved for the treatment of metastatic melanoma in 1998 [1]. Currently, it is the only FDA-approved regimen associated with continuous complete remission over long-term followup (>5 years) [2]. Despite the promise of cure, the overall response rate is grim, at 16% [3]. HD IL-2 therapy requires hospitalization with administration via central venous access and is associated with significant toxicities including capillary leak syndrome, cardiac tachyarrhythmias, and seizures. Because of these risks, its use has been restricted to a select group of patients with excellent performance status. Patients with melanoma brain metastases are usually considered to be ineligible for HD IL-2 therapy due to concerns of life-threatening cerebral edema and neurotoxicity. At our institution, patients with melanoma brain metastases, who were suitable candidates by all other criteria, were considered eligible for treatment. Our aim was to describe our institution's experience of HD IL-2 therapy in melanoma patients with brain metastases.

## 2. Materials and Methods

A retrospective chart review was performed on all melanoma patients with brain metastases treated with HD IL-2 therapy (720,000 IU/kg per dose intravenously; 14 doses, 2 cycles per course, maximum 2 courses) from January 1999 to June 2011 at Saint Louis University. Treatment was administered on an oncology inpatient ward with telemetry monitoring.

Data points including demographics, intracranial and extracranial sites of metastases, type and timing of treatments for brain metastases, and number of brain metastases were recorded. Global response (response of both intracranial and extracranial disease) was measured using RECIST criteria v1.1. In the assessment of extracranial response, the classification of “mixed response” (shrinkage or resolution of some lesions with growth of other lesions or development of new lesions) was also used.

Two-time estimates of survival were documented: length of survival from diagnosis of melanoma brain metastases and length of survival from initiation of HD IL-2 treatment. 

The disease-specific graded prognostic assessment (DS-GPA) scores were calculated for each patient using the patient's Karnofsky performance status (KPS) score and number of brain metastases [4]. The predicted duration of survival of DS-GPA score was compared to actual duration of survival after diagnosis of melanoma brain metastases and actual survival after HD IL-2 initiation.

Adverse effects and toxicity were classified according to common terminology criteria for adverse events (CTCAE) Version 4.

## 3. Results

Eight patients who underwent HD IL-2 therapy had brain metastases prior to treatment. There were 5 men and 3 women within this cohort, and the median age was 52.2 years (26.8–61.1 years). Seven patients had stable brain metastases (defined as metastatic brain lesions that did not require steroids nor cause neurological deficits) at the start of treatment. Patient 3 underwent radiosurgery to remove a solitary brain lesion prior to HD IL-2 treatment. This patient's brain lesion demonstrated complete radiosurgical response (i.e., did not show any evidence of melanoma brain metastasis on imaging) prior to treatment, and the patient started HD IL-2 with metastatic lung lesions.

### 3.1. Extracranial Sites of Metastasis

All 8 patients had extracranial metastasis at the time of diagnosis of melanoma brain metastasis. Seven of 8 patients had extracranial metastasis at the start of HD IL-2 treatment. One patient (Patient 2) had metastatic bowel lesions completely resected prior to HD IL-2; at the start of treatment, this patient had a solitary brain lesion. Three patients had diffuse disease including visceral metastases; two of 3 of these patients also had bone metastases. Four patients had extracranial disease limited to subcutaneous/muscle tissue and/or lungs at the start of treatment. (see Table 1).

### 3.2. Overall Survival and Global Disease Response

Median overall survival for the entire study cohort (*n* = 8) was 8.7 months (2.1 to 19.0 months). The best response was observed in Patient 3, the patient who started HD IL-2 therapy with metastatic disease limited to the lungs only (after complete radiosurgical response of a solitary brain lesion prior to treatment). This patient experienced marked partial response to HD IL-2 therapy, underwent resection of residual lung lesions, and was deemed to show surgical complete response in this analysis. All patients with melanoma brain metastases at first dose (*n* = 7) showed progression on global disease response assessment. Median overall survival was 6.7 months (range 2.1–18.2 months) for this group. 

### 3.3. Intracranial versus Extracranial Response

One patient, Patient 6, had findings suggestive of intracranial response on posttreatment imaging evaluation. 

Patient 3 had extracranial disease only at the start of treatment (after complete radiosurgical response of a solitary brain lesion); the patient's systemic disease showed marked partial response. Patient 2 had solely intracranial disease when starting HD IL-2 after metastatic bowel disease was resected prior to HD IL-2 initiation. This patient's intracranial disease progressed.

In 6 patients who had both brain and extracranial disease at the start of HD IL-2, extracranial mixed response was seen in 2 patients (Patient 5 and Patient 6), and extracranial stable disease was seen in 1 patient (Patient 4). For the other 3 patients with documented systemic progression by imaging, one patient (Patient 8) had documented intracranial progression. Brain imaging for the other 2 patients with documented systemic progression (Patient 1 and Patient 7) was not performed. This is denoted as “undocumented” intracranial progression in Table 1.

### 3.4. Disease-Specific Graded Prognostic Assessment Score (DS-GPA) and Survival

The DS-GPA scores were calculated for each patient using the Karnofsky performance status (KPS) score and number of brain metastases. DS-GPA scores of 1 and 4 were observed in one patient each. Four patients had DS-GPA of 2, and 2 patients had DS-GPA of 3. See Table 2.

For the majority of patients, both the survival after diagnosis of melanoma brain metastasis and survival after HD IL-2 initiation were longer when compared to the predicted median duration of survival based on the patient's DS-GPA score. (see Table 2). Survival after diagnosis of melanoma brain metastasis for the majority of patients (7 of 8) was also longer than predicted duration of survival based on DS-GPA score. In one case, Patient 7, the duration of survival (5.1 months) was less than predicted compared to the patient's corresponding DS-GPA score of 3 (8.77 months). In 6 of 8 patients, the survival after HD IL-2 initiation was longer than predicted based on DS-GPA score.

### 3.5. Chronology and Timing of Therapy

**Figure 1:**
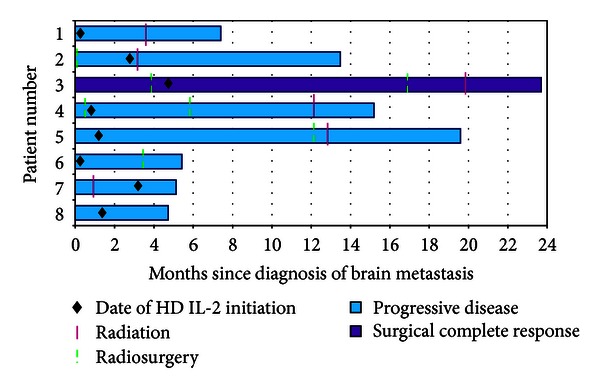
Chronology of therapy for melanoma brain metastases.

Information regarding chronology and timing of therapy from the day of metastatic melanoma brain disease is seen in Figure 1. Three patients had radiosurgery, and 1 patient had whole-brain radiation prior to starting HD IL-2 therapy. After treatment with HD IL-2, 5 patients received consolidation whole-brain radiation, and 4 patients received radiosurgery.

### 3.6. Number of Brain Metastases and Survival

**Figure 2:**
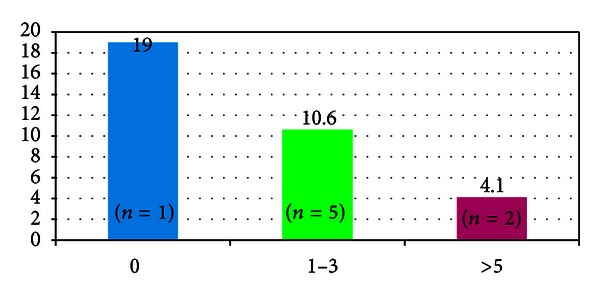
Number of discrete brain metastases at the start of treatment and median overall survival (months).

Most patients (*n* = 5) had 1–3 discrete brain lesions at the start of HD IL-2 therapy lesions. The size of these lesions ranged from 1 to 2.5 cm. Median overall survival for patients with 1–3 was 10.6 months. Two patients had several (>5) small (<1 cm) brain lesions, one with 7 lesions and the other with 10 lesions. Median survival for this group was 4.1 months. The survival of the patient whose brain metastasis was resected prior to first dose of HD IL-2 was 19.0 months. (see Figure 2).

### 3.7. Side Effects

**Figure 3:**
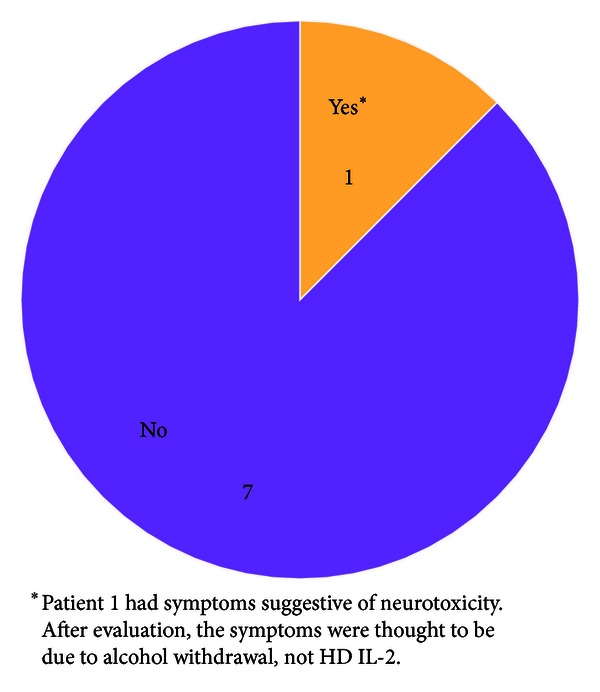
Symptoms suggestive of neurotoxicity during HD IL-2 treatment.

One patient (Patient 1) had symptoms during treatment (mental status changes and tremors) that were suggestive of neurotoxicity (Figure 3). During admission, it was revealed that this patient had a history of alcohol abuse. The neurological symptoms improved when the patient was put on an alcohol withdrawal protocol, and HD IL-2 therapy was restarted and completed. In two other patients, transitory episodes of delirium/confusion of Grade 2 severity were noted.

Grade 3 events observed are listed in decreasing frequency with number of incidents as follows: oliguria (6), tachycardia (2), and hypoxia (1). Other adverse effects observed during treatment include elevated troponins, hypertension/hypotension, fevers/chills, rigors, rashes, pruritus, upper and lower extremity swelling, weight gain, thrombocytopenia, diarrhea, and transaminitis. One patient's (Patient 3) hospital admission was complicated by a pulmonary embolism, which was not life-threatening.

## 4. Discussion

Retrospective studies estimate the cumulative incidence of brain metastasis in melanoma patients to be <10% [5]. In advanced melanoma, the estimated incidence increases from one-third to one-half of patients, but it may actually be much higher [6, 7]. In a review of autopsies of 216 patients with advanced melanoma, brain metastases were identified in ~75% of patients [8]. This suggests that metastatic brain disease may be missed with current imaging modalities or that available treatments may not effectively cross the blood-brain barrier or treat metastatic brain disease. 

With existing treatment options, median overall survival for patients with melanoma brain metastases is approximately 4 months [9]. Radiation and surgical therapy have been considered as the mainstay of treatment [10]. Systemic therapeutic options are limited for these patients, and they have historically been excluded from clinical trials. The literature is limited regarding the use of HD IL-2 in patients with melanoma brain metastases. In this observational study, all patients received radiotherapy or stereotactic radiosurgery before or after HD IL-2 therapy. All previous reports have described patients who had received HD IL-2 with untreated melanoma brain metastases, except for one case in which the patient completed whole brain radiation prior to HD IL-2 treatment [11, 12]. 

The best response and the longest survival rate after HD IL-2 initiation in this study cohort (19.0 months) were observed in the patient whose solitary brain lesion showed complete radiosurgical response prior to treatment (Patient 3). One factor that likely contributed to this patient's prolonged survival was that this patient started HD IL-2 therapy with only one site of distant metastasis (lungs). In this case, radiosurgery decreased the overall systemic tumor burden by eliminating the patient's other site of distant metastasis (brain). While radiosurgery for melanoma brain metastasis, as a means to decrease systemic tumor burden, has not been specifically studied, it has been proposed that surgical resection may have immediate therapeutic benefits in Stage 4 disease by decreasing the effects of tumor-induced immunosuppression [13]. Surgical resection may also have direct beneficial effects on the body either through the disruption of the metastatic cascade that leads to hematogenous seeding or by triggering the enhancement of the patient's own immune system. In support of these theories is that Patient 2 also had a long duration of survival (13.5 months). Patient 2 also started HD IL-2 with one site of distant metastasis (brain) after complete resection of another site of distant metastasis (small bowel). 

Surgical resection, as one component of Stage 4 disease management, has been associated with favorable outcomes. In a large retrospective review of patients who developed distant metastases during the first Multicenter Selective Lymphadenectomy Trial (MSLT-I), Howard et al. demonstrated that surgical treatment (alone or in combination with other treatment modalities) in Stage 4 melanoma disease was associated with improved survival for any M category when compared to patients who received systemic treatment alone [13]. In the subset analysis of patients with M1c patients, the authors found that median duration of survival was significantly longer (15.0 months) for those who had received both surgery and systemic therapies when compared to M1c patients who received systemic therapy alone (6.3 months). While this analysis did not include a subanalysis of M1c patients with brain disease, it seems likely that complete resection of brain disease or complete radiosurgical response would have a positive effect on outcome. In addition, it is possible that the eradication of metastatic brain disease (an anatomic site associated with poor prognosis) may offer a greater impact on survival compared to eradication of disease at other systemic sites.

Two other patients (Patient 2 and Patient 4) were treated with radiosurgery to remove brain lesions prior to HD IL-2 therapy. While complete radiosurgical response of the metastatic brain disease was not achieved, in part due to metastatic brain lesion location, these patients also had survival >12 months after diagnosis of melanoma brain metastasis. This may suggest that attempting to decrease intracranial tumor burden, even if not complete, may be of benefit to patients with melanoma brain metastasis as well. 

When evaluating the findings in our cohort compared to previous reports of patients who received radiosurgery before immunotherapy, the survival rates in this study were similar. Liew et al. performed a large retrospective review of 333 patients with melanoma brain metastasis who underwent stereotactic surgery to identify clinical factors and treatments associated with extended survival [14]. As in our findings, Liew et al. found that patients with a single brain lesion controlled extracranial disease, and treatment with immunotherapy had the longest survival. The duration of survival of Patient 3 (19.0 months) is comparable to the median duration of survival of patients who had this same disease course (22.0 months). In addition, median duration of patients who received both radiosurgery and immunotherapy in our cohort (15.2 months) was similar to that found in the analysis of Liew et al. (13.8 months).

Some prognostic factors that have been identified to affect survival for patients with melanoma brain metastases—Karnofsky performance status (KPS) score and number of brain metastases—were used to develop the disease-specific graded prognostic assessment score (DS-GPA). The DS-GPA has been validated as a measure that can be used to predict duration of survival after diagnosis of melanoma brain metastasis [4]. In our cohort of patients, duration of survival tended to correlate with DS-GPA score; patients with higher DS-GPA scores had longer survival. In all but one case, the duration of survival of patients in this cohort was longer than the survival predicted by the patient's corresponding DS-GPA score. Additionally, the duration of survival after HD IL-2 initiation was longer than predicted by DS-GPA score in 6 out of 8 cases. Notably, the median duration of survival of patients in this cohort with DS-GPA score of 2 (11.3 months) was much longer than predicted (4.7 months). In this analysis, there was one anomaly, Patient 7's duration of survival after metastatic melanoma brain diagnosis, 5.1 months, was shorter than that predicted by the patient's DS-GPA score of 3 (8.77 months). It is difficult to make conclusions from this small sample, but HD IL-2 did not appear to negatively affect patients' survival.

Our study was limited by its retrospective nature, which precluded accurate collection of data regarding systemic and intracranial progression. Often patients did not undergo brain imaging to assess intracranial response if systemic progression was seen at followup. At least one patient (Patient 6) had documented intracranial response; this patient's systemic disease exhibited mixed response. One other patient had extracranial mixed response, and 2 patients showed extracranial partial response. This may suggest that even if HD IL-2 does not lead to intracranial response in patients, it may be of benefit to patients with melanoma brain metastasis by treating their systemic disease. 

The results of the safety assessment are perhaps the most significant findings from the analysis of this study cohort. Of note, there did not seem to be an increased morbidity associated with treating patients with melanoma brain metastasis with HD IL-2 compared to treating those without a history of melanoma brain metastasis. The frequency and severity of adverse events did not seem to be higher than that observed in our institutional experience or reported in the package insert, and there were no unique events identified. There was one patient (Patient 1) who displayed signs that were suggestive of neurotoxicity. After evaluation, the symptoms were thought to be due to alcohol withdrawal since the symptoms improved on an alcohol withdrawal protocol, and HD IL-2 was able to be completed. In fact, this patient had a survival of 6.7 months after HD IL-2 initiation, which was longer than the duration of survival predicted by the patient's DS-GPA score of 2 (4.7 months). Neurological symptoms observed in 2 other patients did not seem to be different than those observed in patients without melanoma brain metastasis treated with HD IL-2. While pulmonary embolism has been reported to be fatal in <1% of patients undergoing treatment with HD IL-2 [1], the patient who had a pulmonary embolism (Patient 3) during treatment actually had the longest survival in this analysis (19.0 months from initiation of HD IL-2 and 23.5 months from diagnosis of melanoma brain metastasis). 

Further studies in larger cohorts of patients are still needed to determine the safety risk and likelihood of response to HD IL-2 in patients with melanoma brain metastases. HD IL-2 may be best utilized as part of a multimodal treatment in patients with melanoma brain metastasis with brain lesion-directed therapy (radiosurgery or radiation) and targeted extracranial disease treatment (resection or chemoembolization) and possibly in combination with other systemic treatments. 

The FDA approval of two new drugs, ipilimumab and vemurafenib, has led to discussion of treatment algorithms for patients. There is preliminary data to suggest that these agents may aid in the treatment of melanoma brain metastasis [15, 16]. The patients in our analysis were treated before ipilimumab and vemurafenib were available. For all patients with advanced melanoma, there is still an ongoing discussion about the best sequence to employ in the treatment of melanoma because of concerns that potential candidates for HD IL-2 may no longer be eligible for treatment due to complications from the preceding therapy. Specifically, the use of ipilimumab and long-term course of steroids to treat ipilimumab immune-related side effects may preclude subsequent therapy with HD IL-2 in a previously eligible patient. 

## 5. Conclusions

While the role of HD IL-2 in melanoma patients with brain metastases has yet to be defined, we believe that brain metastases should not be considered an absolute contraindication to therapy, but multiple disease and patient factors must be considered when deciding whether or not to initiate treatment. While it is difficult to draw definitive conclusions from this small cohort of patients, our data is informative from the perspective that these patients received HD IL-2 therapy in a safe fashion without treatment-related mortality and suggests that some patients may be treated without development of neurotoxicity. Further studies are needed to improve management of this cohort of patients which are known to have poor prognosis. In addition to recent studies on ipilimumab [17] and vemurafenib, promising data from trials on programmed death-1 (PD-1) inhibitors are forthcoming [18]. Until the response of other treatments can be further clarified, HD IL-2 should be considered as a treatment option in patients with melanoma brain metastases who are otherwise eligible for therapy.

## Figures and Tables

**Table 1:** Patient characteristics (sites of metastasis, disease response, and duration of survival).

Patient	No. of brain lesions at diagnosis of brain metastases	Extracranial sites of metastasis	Intracranial response	Extracranial response	Global response	Duration of survival from HD IL-2 initiation (months)	Duration of survival from diagnosis of brain metastases (months)
1	2	Subcutaneous lesions	Undocumented progression	Progression	Progression	6.7	7.4
2	1	None at start of HD IL-2 (small bowel mets s/p resection)	Progression	n/a	Progression	10.6	13.5
3	1	Lungs	n/a (complete radiosurgical response of solitary brain met)	Partial response (residual lung lesions resected)	Surgical complete response	19.0	23.7
4	3	Muscles, retrocrural soft tissue	Progression	Stable disease	Progression	13.3	15.2
5	3	Pulmonary soft tissue, LNs, liver, kidney, prostate, and bones	Progression	Mixed response	Progression	18.2	19.6
6	15–20	Lung (multiple), paraspinal muscles	Partial response	Mixed response	Progression	5.3	5.4
7	1	Lung (multiple), mediastinum, soft tissue, kidney, spleen, LNs, and bones	Undocumented progression	Progression	Progression	2.1	5.1
8	7	Hilum, lungs, pulm. soft tissue, and adrenal glands	Progression	Progression	Progression	3.0	4.7

**Table 2:** Predicted duration of survival versus actual duration of survival (by DS-GPA score).

Patient	DS-GPA	Predicted median duration of survival based on DS-GPA (months)	Duration of survival from HD IL-2 initiation (months)	Duration of survival from diagnosis of brain metastases (months)
8	1	3.38	3.0	4.7

1	2	4.7	6.7	7.4
4	2	4.7	13.3	15.2
5	2	4.7	18.2	19.6
6	2	4.7	5.3	5.4

2	3	8.77	10.6	13.5
7	3	8.77	2.1	5.1

3	4	13.2	19.0	23.7
